# Motor Simulation and the Bodily Self

**DOI:** 10.1371/journal.pone.0017927

**Published:** 2011-03-25

**Authors:** Francesca Ferri, Francesca Frassinetti, Marcello Costantini, Vittorio Gallese

**Affiliations:** 1 Department of Neuroscience, University of Parma, Parma, Italy; 2 Department of Psychology, University of Bologna, Bologna, Italy; 3 Department of Neuroscience and Imaging, University G. d'Annunzio, Chieti, Italy; 4 Institute for Advanced Biomedical Technologies (ITAB), Foundation University G. d'Annunzio, Chieti, Italy; 5 Brain Center for Social and Motor Cognition, Italian Institute of Technology, Parma, Italy; The University of Melbourne, Australia

## Abstract

Previous studies demonstrated the human ability to implicitly recognize their own body. When submitted to a visual matching task, participants showed the so-called self-advantage, that is, a better performance with self rather than others' body or body parts. Here, we investigated whether the body self-advantage relies upon a motor representation of one's body. Participants were submitted to a laterality judgment of self and others' hands (Experiment 1 and 3), which involves a sensory-motor mental simulation. Moreover, to investigate whether the self-advantage emerges also when an explicit self processing is required, the same participants were submitted to an explicit self-body recognition task (Experiment 2). Participants showed the self-advantage when performing the laterality judgment, but not when self-recognition was explicitly required. Thus, implicit and explicit recognition of the bodily self dissociate and only an implicit recognition of the bodily self, mapped in motor terms, allows the self-advantage to emerge.

## Introduction

Neuropsychological and neuroimaging studies show that the body is a “unique” object. Indeed, specific brain structures are involved in the visual processing of the human body [1], [2], [3]. Viewing non-facial body parts selectively activates a lateral occipito-temporal cortex (OTC), called extrastriate body area (EBA), and an area located in the fusiform gyrus, known as fusiform body area (FBA) [3], [4], [5]. Moreover, a topographically organized body part map has been described within the OTC, with distinct clusters of voxels showing clear preference for different visually presented body parts [6]. In line with this evidence, a hand-selective region has been recently revealed in the left lateral occipital sulcus, partially overlapping with EBA, which could be functionally and anatomically dissociated from it [7].

When processing a human body, a critical distinction can be made between one's own body and the body of others [8], [9]. Studies using different methods (behavioral, fMRI, TMS studies) have shown that the recognition of “self body” is independent from the recognition of other people's bodies. Interestingly, self-related body stimuli are processed faster and more accurately compared to other-related body stimuli (self-advantage, see [10], [11]). This advantage for self body processing was revealed by a visual task in which an explicit self body recognition was not required. Participants were submitted to a matching task in which three pictures, representing a body part (hand, foot, arm and leg), were presented vertically aligned at the centre of the computer screen. They were asked which of the two stimuli, the upper or the lower one, matched with the central target. Participants' performance was more accurate when one of the stimuli belonged to them compared to when they belonged to someone else.

The mechanism supporting the body self-advantage is still under debate. One hypothesis is that bodily self recognition is based on a sensory-motor representation (for a review, see [12]).

The main aim of the present study is to shed new light on the implicit body self-advantage. To this purpose, we investigated the contribution of visuo-motor body representation with two different tasks. In a first experiment (Experiment 1) healthy participants were submitted to a laterality judgment task with either self or others' hands as body stimuli. In a second experiment (Experiment 2) we employed the same stimuli as in Experiment 1, but asked participants to explicitly recognize their own hand. Finally, in a third control experiment (Experiment 3) we ruled out the possibility that the results of the first experiment were simply driven by any sort of familiarity of “priming” effects.

In the laterality judgment task (Experiments 1 and 3) participants were requested to report the laterality (left or right) of depicted body parts presented in different angular orientations. We adopted this task because it is well known that in order to perform it participants simulate a motor rotation of *their own body parts* so as to match that of the *observed stimulus* [13], [14]. Mental motor rotation of body parts shares the same temporal and kinematic properties with actual body rotation in space [13], [15], [16], [17], [18], [19]. This idea is further corroborated by evidence showing that longer mental rotation times are needed for stimuli orientations corresponding to body part positions difficult to be maintained [13], [20], [21]. Since previous studies [22], [23] suggest that the left-right judgment of body parts relies upon the visuo-motor representation of one's own body, we hypothesize that the laterality judgment in Experiments 1 and 3 should be easier when the displayed stimulus is one's own hand. Indeed, only in this case, the displayed stimulus matches with the mentally rotated hand (self-advantage). If this is true, the visuo-motor representation of one's own body is crucial for the self-advantage.

Interestingly, the self-advantage described in previous studies [10], [11], [24] has been found without requiring an explicit self body recognition, as it emerged on the basis of a mere implicit self-body recognition. As a consequence, the explicit recognition of one's own body does not seem to be necessary for the emergence of the self-advantage. To address the question of whether the requirement of explicitly recognizing one's own body is a sufficient condition for the emergence of the self-advantage, we ran a second experiment using the very same stimuli of the Experiment 1. Here (Experiment 2), participants were asked to explicitly recognize the identity of the displayed hand, that could be either the participants' or other people's hands. If the requirement of explicit self recognition is a sufficient condition for the self-advantage, this should be found also in the Experiment 2. Alternatively, a dissociation between implicit and explicit self body processing should be found.

## Methods

### Participants

Twenty-four right-handed healthy participants (mean age = 37,5 years; range 20–55), naive as to the purpose of the study, participated in each experiment. The same participants (12 men and 12 women) took part in Experiment 1 and Experiment 2. A different group of participants (14 men and 10 women) took part in Experiment 3. Participants had no history of neurological diseases as self reported. All participants gave their written informed consent for participation in the study. The experimental protocol was approved by the Ethics Committee of the University of Parma.

### Stimuli and Procedure

**Figure 1:**
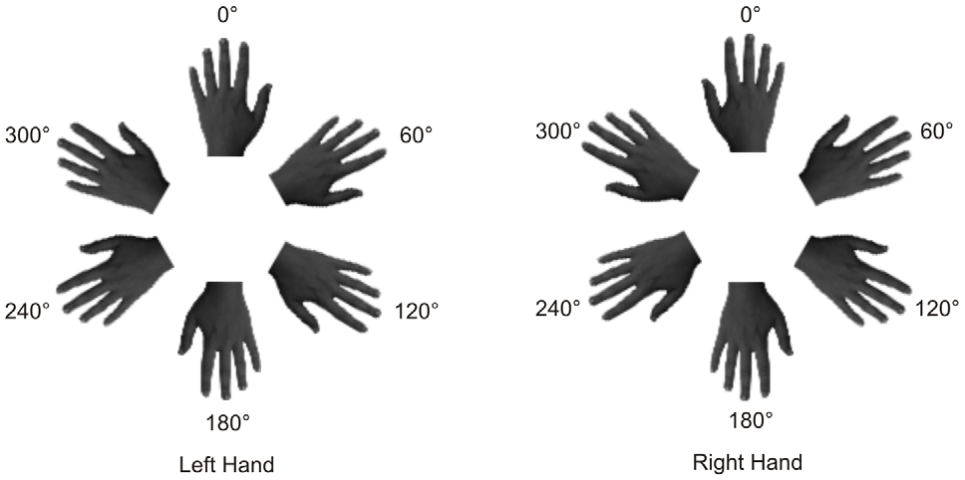
Stimuli. Experimental stimuli consisted of pictures depicting the dorsal view of right and left hands in six different clockwise orientations. Images of participant's hands or of three other people's hands were presented one at a time in ‘self’ trials and ‘other’ trials, respectively.

The experimental stimuli consisted of grey-scale pictures of the dorsal view of right and left hands (see Figure 1). The hands of each participant were photographed with a digital camera in a session prior to the experiments (1 week before). This session took place in a controlled environment with constant artificial light and a fixed distance between the camera lens and the hands (40 cm), which were always photographed in the same position. Subsequently, photographs were modified with Adobe Photoshop software: they were cut from the original picture, pasted on a white background, and reoriented into the different rotated positions. Other people's hands were selected from this database as the best match for size, skin color, age, and gender, in comparison with each participant's hands. The sizes of the hands were compared in the pictures, in order to minimize the differences between matched hands both in length and in width. In addition, the ages of the people whose hands were matched with the participants' hands varied within 0 to 3 years of the participants' ages.

Images of hands were presented one at a time at the centre of the computer screen in six different clockwise orientations from the upright (0°, 60°, 120°, 180°, 240°, 300°). The upright orientation was defined as fingers pointing upwards.

Stimuli depicted the participant's own left or right hand in half of the trials (‘self’ trials). In the other half of the trials, stimuli depicted the right or left hand of other three people (‘other’ trials, Experiments 1 and 2). In Experiment 3 stimuli presented in the ‘other’ trials depicted the right or left hand of only one other individual. This methodological change was done to control for “priming” or familiarity effects that might occur in the laterality judgment task.

Participants sat in front of a PC screen, at a distance of about 30 cm. Stimuli presentation was controlled by E-Prime (Psychology Software Tools Inc., [25], [26]). Each trial started with a central fixation cross (500 ms duration), followed by stimulus presentation. The trial was timed-out as soon as participants responded (up to 4000 ms).

In Experiment 1 and 3 participants were required to judge the laterality (left or right) of observed digital images of hands by pressing as accurately as possible and within the allowed time interval, a left or a right response key, with their left and right index fingers, respectively.

In Experiment 2, participants were required to explicitly judge whether the displayed hand corresponded or not to their own hand by pressing as accurately as possible and within the allowed time interval, a left or a right previously assigned response key, with their left and right index fingers, respectively. The response keys were counterbalanced between subjects.

Each Experiment consisted of 288 trials, 72 trials for each of the four conditions: self-right, self-left, other-right, other-left. In particular, in Experiment 1 and 2 the self right and left hand stimuli were shown to participants 72 times each; others' right and left hand stimuli were shown only 24 times. To rule out the possibility that higher repetition rates of self, compared to others' stimuli led to a “priming” effect during the laterality judgment task, a control Experiment 3 was performed. In this experiment others' right and left hands belonged to only one “other” individual. Thus, self and others' right and left hands were shown 72 times each. In all the three experiments, each orientation was randomly depicted 12 times per condition. Experiments were always preceded by a task-specific practice block.

Since Experiment 1 investigated the implicit and Experiment 2 the explicit self-bodily recognition, Experiment 1 was always conducted before Experiment 2. The same group of participants performed both Experiments in one single session. Experiment 3 was administered in a separate session to a different group of participants.

## Results

### Results of Experiment 1

**Figure 2:**
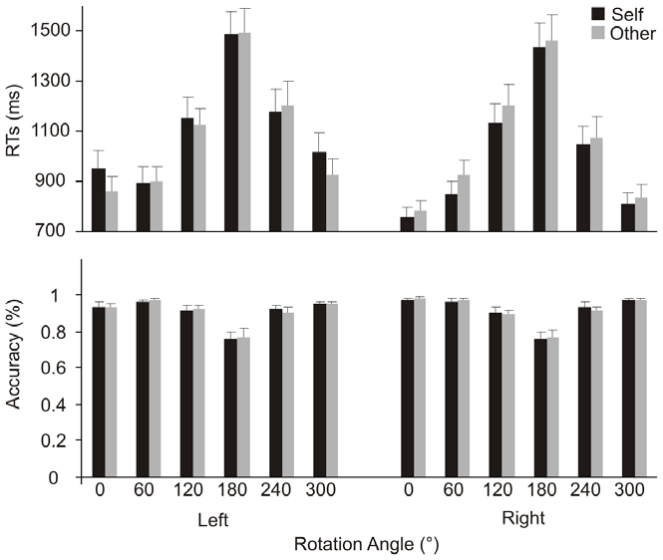
Experiment 1. Mean response times (upper panel) and accuracy (bottom panel) at the different self’ and others' hands stimuli orientations in the Implicit task. Error bars depict the standard error of the mean.

Data are shown in Figure 2. To test the presence of self-advantage with the laterality judgment task, an ANOVA was conducted on participants' reaction times (RTs), with Owner (one's own and other people's stimuli), Laterality (left and right), and Orientation (0°, 60°, 120°, 180°, 240° and 300°) as within-subject factors. The Newman-Keuls test was used for all post-hoc comparisons.

The ANOVA revealed the significance of the main effect of Laterality [F(1,23) = 9.28, p<.006, η_p_^2^ = .29], since RTs to right stimuli were faster than RTs to left stimuli (1028 ms vs 1100 ms). The factor Orientation was also significant [F(5,115) = 57.74, p<.001, η_p_^2^ = .72]. This effect was accounted for by faster RTs at 0°, 60° and 300° (839, 893, 898 ms, respectively) compared to RTs at 120°, 180°, 240° (1155, 1472, 1128 ms, respectively; p<.001 in all cases). The Laterality by Orientation interaction was also significant [F(5,115) = 4.01, p<.002, η_p_^2^ = .15], because of the faster performance with right than left stimuli at 0° (771 ms vs. 908 ms), 240° (1064 ms vs.1192 ms), and 300° (822 ms vs. 974 ms, p<.01 for all comparisons). Relevant to the main goal of the study, the interaction Owner by Laterality was also significant [F(1,23) = 5.82, p<.02, η_p_^2^ = .20]. The interaction was explained by faster RTs to right self stimuli compared to right others' stimuli (1007 ms vs. 1048 ms, p<.05, see Figure 2). No significant difference was observed for left hands between self and others' stimuli (1114 ms vs. 1087 ms, p = .19). Moreover, RTs to self-right stimuli were faster than RTs to self-left ones (and other-left; p<.002 for all comparisons), whereas only a trend to significance was found between other-right and other-left stimuli (p = .07).

When the same analysis was conducted on accuracy (percentage of correct responses), only the factor Orientation was significant F[(5,115) = 20.2, p<.0003, η_p_^2^ = .47], being subjects less accurate at 180° (76%) than at all other orientations (0° = 96%, 60° = 97%, 120° = 91%, 240° = 92%, 300° = 96%, p<.0001 for all comparisons). The other orientations were not significantly different.

### Results of Experiment 2

**Figure 3:**
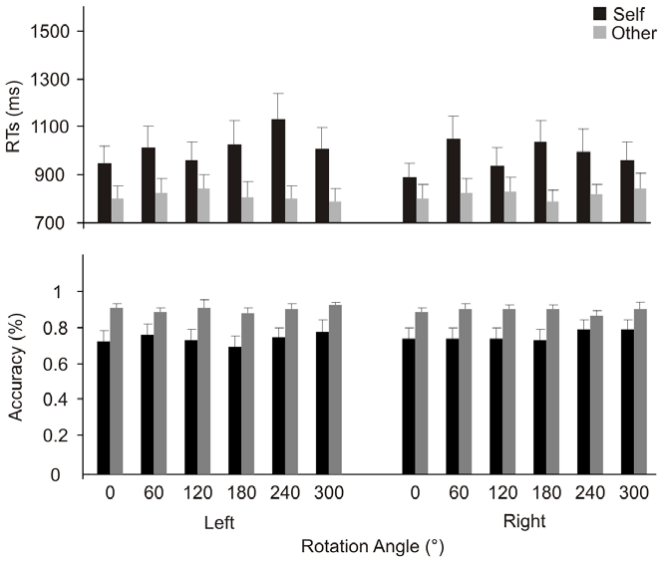
Experiment 2. Mean response times (upper panel) and accuracy (bottom panel) at the different self’ and others' hands stimuli orientations in the Explicit task. Error bars depict the standard error of the mean.

Data are shown in Figure 3. An ANOVA similar to that of Experiment 1 and 2 was conducted on participants' reaction times (RTs), with Owner, Laterality and Orientation as within-subject factors.

The factor Owner was significant F[(1,23) = 18.66, p<.001, η_p_^2^ = .45], since participants responded faster to others' than to self stimuli (814 vs 997 ms, see Figure 3). No other significant effects were found.

The same analysis conducted on accuracy (percentage of correct responses) confirmed a worse performance with self than with others' stimuli (76% vs 91%, F[(1,23) = 11.29, p<.001, η_p_^2^ = .33]).

### Results of Experiment 3

To rule out the possibility that the presence of the self-advantage for right hands with the laterality judgment task was due to any sort of familiarity or “priming” effect, we asked a new group of participants to perform the same task in the control Experiment 3. In this experiment, each self and other's stimulus was shown the same number of times. An ANOVA was conducted on participants' reaction times (RTs), with Owner (one's own and other people's stimuli), Laterality (left and right), and Orientation (0°, 60°, 120°, 180°, 240° and 300°) as within-subject factors. The Newman-Keuls test was used for all post-hoc comparisons.

The ANOVA revealed the significance of the main effect of Laterality [F(1,23) = 6.1, p<.05, η_p_^2^ = .21], since RTs to right stimuli were faster than RTs to left stimuli (838 ms vs 867 ms). The factor Orientation was also significant [F(5,115) = 23.9, p<.001, η_p_^2^ = .86]. This effect was accounted for by faster RTs at 0°, 60° and 300° (704, 755, 732 ms, respectively) compared to RTs at 120°, 180°, 240° (888, 1165, 870 ms, respectively; p<.001 in all cases). Most interestingly, the significance of the interaction Owner by Laterality, found in Experiment 1, was confirmed in the present control experiment [F(1,23) = 4.5, p<.05, η_p_^2^ = .16]. Once again this interaction was explained by faster RTs to right self stimuli compared to right others' stimuli (831 ms vs. 844 ms, p<.05). No significant difference was observed for left hands between self and others' stimuli (868 ms vs. 865 ms, p = .55). Moreover, RTs to self-right stimuli were faster than RTs to self-left ones (and other-left; p<.0002 for all comparisons). Similarly, RTs to other-right stimuli were faster than RTs to other-left ones (and self-left; p<.002 for all comparisons).

When the same analysis was conducted on accuracy (percentage of correct responses), only the factor Orientation was significant F[(5,115) = 14.8, p<.001, η_p_^2^ = .60], being participants less accurate at 180° (86%) than at all other orientations (0° = 97%, 60° = 97%, 120° = 95%, 240° = 96%, 300° = 97%, p<.001 for all comparisons). The other orientations were not significantly different.

## Discussion

In this study we investigated whether and to what extent the so-called self-advantage [10], [11], [24] is based on a motor simulation. To this aim healthy participants were submitted to a hand laterality judgment task. Crucially, the hand to be judged could be either the participants' own hand or other people's hand. Results showed an advantage when judging one's own right compared to others' hands. Such an advantage was reflected by faster reaction times when responding to the former stimulus compared to the latter ones (Experiment 1 and 3). It is worth noting that this advantage was present in a task in which explicit self recognition was not required. By contrast, the self advantage was lacking in the second experiment where self recognition was explicitly required. Indeed, a worse performance with self-related stimuli compared to other-related stimuli was observed.

Experiments 1 and 3 differed from Experiment 2 with respect to two main variables. The first one is the motor strategy required to solve the task, present in the laterality judgment task (Experiment 1 and 3), but not in the self-body recognition task (Experiment 2). In order to perform the laterality judgment task a mental motor rotation of body parts is required [22], [13], [18], [14]. Coherently, the classical bell-shaped function of RTs found for this task (see Figure 2) constitutes the behavioral signature of mental rotation. On the other hand, the absence of such a function in the RTs of the self-body recognition task (see Figure 3) shows that a motor simulation is not required to accomplish the explicit task. For these reasons the presence of the self-advantage in Experiment 1 and 3, and its absence in Experiment 2 suggest that the bodily self is ultimately linked to a motor representation.

The second variable is the requirement to explicitly recognize self stimuli, which characterizes the second, but not the first and the third experiments. Our data demonstrate that the request of an explicit recognition of one's own body does not lead to the emergence of the self-advantage. Thus, explicit body processing is *per se* neither necessary nor sufficient to grant the body self-advantage.

We are aware the two tasks required two different responses, thus they cannot be directly compared to each other. However, to the best of our knowledge, this is the first study investigating the implicit and explicit self bodily knowledge by means of the very same stimuli and the same experimental procedure. The idea of a dissociation between implicit and explicit self body processing is in agreement with the large amount of neuropsychological studies showing that brain damaged patients can be impaired in explicit while sparing implicit processing. A typical clinical condition in which implicit and explicit processes are dissociated is neglect. Neglect patients fail to explicitly detect stimuli presented in the contralesional affected field. However, they can implicitly process the same stimuli up to the semantic level [27], [28]. Regarding the bodily self, such dissociation is in agreement with the independence of implicit from explicit self-body processing reported by infancy research. Indeed, during development an implicit sense of self and the ability to discriminate self from others appears to emerge earlier than the ability to explicitly self-recognize [29], [30].

Taken together, data from Experiments 1 (confirmed by Experiment 3) and 2, although not directly comparable to each other, suggest that the crucial element for the self-advantage to emerge is the recruitment of a motor simulation. This interpretation is in agreement with and provides a coherent explanation to a variety of previous studies. Tsakiris et al. [31] carried out a study in which participants had to decide whether they viewed their own right hand or someone else's right hand covered with identical gloves, while experiencing a passive displacement of their own right index finger, either generated by the experimenter or by participants' own left hand. The results showed that the performance was significantly better when the displacement of participants' right index finger was self-generated. As argued by Tsakiris, Schutz-Bosbach, & Gallagher [32], this shows that “Self recognition was significantly more accurate when subjects themselves were the authors of the action” (p. 654–655). Coherently, visual and motor related information converge within the OTC in a body part specific manner [8], and the feeling of ownership of the hand positively correlates with activity in the premotor cortex [33].

In a behavioral study Loula, Prasad, Harber, & Shiffrar [34] asked participants to perform a self identification task while observing sagittal displays of point-light depictions of themselves, their friends, and strangers while performing various actions. They found higher sensitivity to one's own motion. Since everyone has little experience of viewing her own body moving, such self-advantage can be easily explained by the activation of observers' own action motor representation. Similarly, a self-advantage was demonstrated by Casile & Giese [35] in a behavioral task, in which only non-visual motor training was available to participants.

The last point to be addressed is the presence of the self-advantage only for participants' right hand. Such selectivity is a further argument in favor of our motor hypothesis of the self-advantage. The presence of the “self-advantage” only for the right hand can be explained by the greater lateralization in hand motor skills observed in right-handers compared to left-handers (e.g., [36]). Neuroimaging studies have shown hemispheric asymmetries in cortical areas associated with body representation in right-handed people, but not in left-handed people. Indeed, right-handed individuals have a greater cortical surface area in the left sensory cortex and stronger activation in the left sensory-motor cortex while performing right hand movements than in the corresponding areas of the right hemisphere. In contrast, left-handed individuals seem to have near-symmetrical surface areas and activations [37], [38], [39]. Similar results have been observed with electroencephalographic (EEG) studies [40], [41]. Furthermore, it was recently shown that right-handers perceive their own right arm and hand as being longer than their left ones, whereas left-handers perceive both arms and hands accurately [42]. Thus, it appears that the conscious perception of the body is grounded on its motor potentialities [43].

Since according to our data the self-advantage relies upon a sensory-motor representation, the presence of the self-advantage only for self right hand stimuli is likely the consequence of the greater involvement of the left, rather than the right, sensory-motor areas in right-handers during a mental motor task. Given such a near-symmetrical cortical representation in left-handers, future studies on this population might help us to shed new light on this phenomenon. Recent data seem to support our hypothesis. Conson and colleagues [44] asked right-handed and left-handed healthy participants to categorize full-colored pictures of hands, presented according to the egocentric or the allocentric perspective, as belonging to themselves or to other people. They found that both right- and left-handers were faster in recognizing dominant hands (right and left hand, respectively) in egocentric perspective, and others' non-dominant hands in allocentric perspective.

Possibly one may argue that the self advantage we found in Experiment 1 can be construed in terms of “priming” effect or any sort of visual familiarity. Indeed, in this experiment self stimuli were presented 72 times while each of the three others' stimuli was presented only 24 times. To deal with this possible concern, we ran a third control experiment in which we used the hands of only one other individual, thus matching the number of occurrences of each stimulus in terms of identity. We found the same results as in Experiment 1. This rules out the possibility that the self advantage is exclusively due to “priming” effects. Regarding visual familiarity, we believe something different might underpin our behavioral effect. Indeed, out of the total of self-related trials, one half involved the presentation of the right hand while the other half involved the presentation of the left hand. It follows that if perceptual familiarity could fully explain our results, it is not clear why our effect was visible only for right hand stimuli. Our idea is also corroborated by a recent study [45] exploring whether symbolic cues, predicting the appearance of one's own or another person's hand could optimize the processing of these stimuli. Results showed a selective attentional effect with one's own hand, but not with someone else's hand. More relevant for the purpose of our study, in a control experiment the authors tested whether this selective attentional effect could be due to the higher perceptual familiarity. Results showed that participants could use the cues to anticipate the appearance of both stimuli, since a behavioral advantage was observed for all valid stimuli, regardless of their degree of familiarity.

In conclusion, our data demonstrate that implicit and explicit recognition of the bodily self dissociate and that, only when bodily self recognition is implicit, a self-advantage does emerge. Since the implicit mechanism recruits a motor simulation, it follows that the bodily self is primarily mapped in motor terms. Indeed, when explicit self recognition is required and different cognitive and/or perceptually-based mechanisms are likely involved, the self-advantage is lacking. The idea of the motor nature of the bodily self is in agreement with previous philosophical intuitions. Merleau-Ponty posited that our body appears to us as an attitude directed towards a certain existing or possible tasks. When referring to the spatiality of the body he claimed: “[…] my body appears to me as an attitude directed towards a certain existing or possible task. And indeed its spatiality is not, like that of external objects […], a spatiality of position, but a spatiality of situation”.
